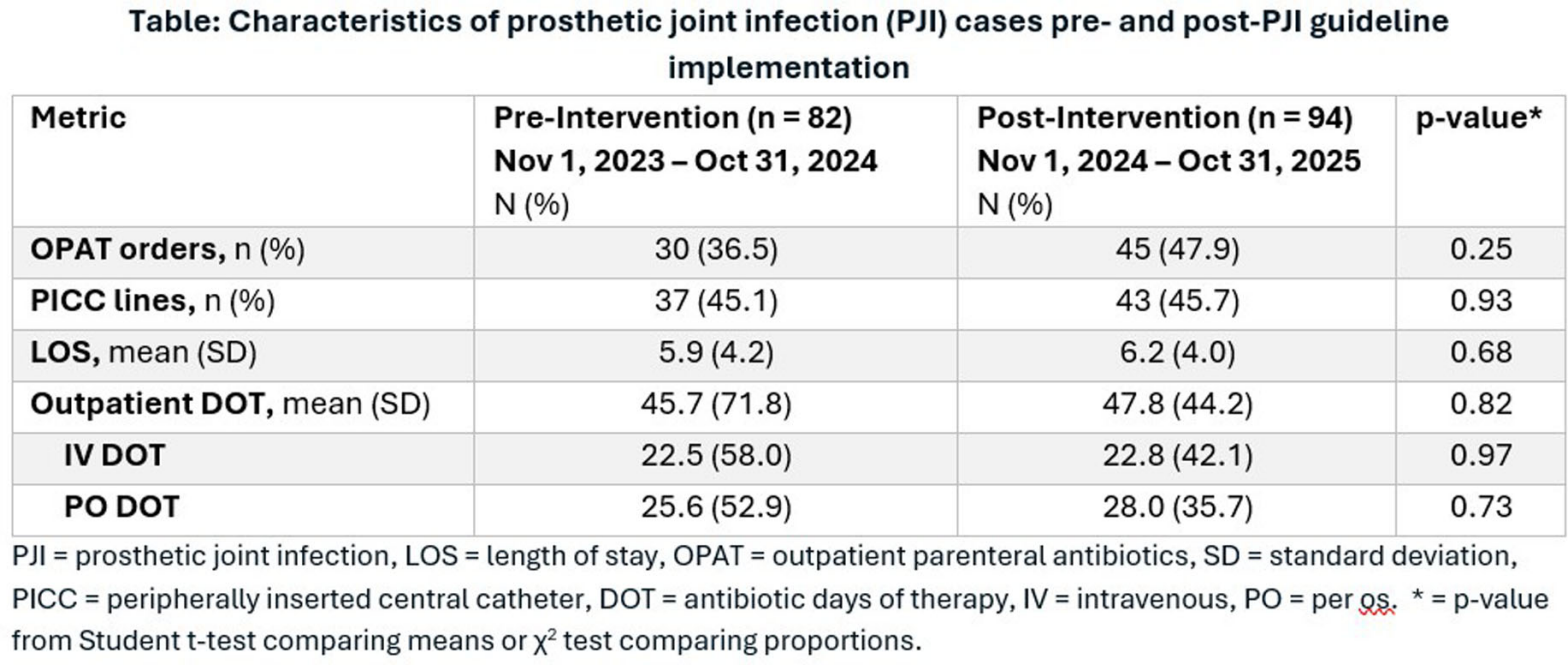# 118 Impact of Obesity on Patient Characteristics and Clostridioides difficile Infection Outcomes in Davidson County, Tennessee, 2016–2023

**DOI:** 10.1017/ash.2026.10532

**Published:** 2026-06-23

**Authors:** Elizabeth Kim, Julianne Gent, Laila Woc-Colburn, Sujit Suchindran, Nirja Mehta

**Affiliations:** 1 Emory University School of Medicine; 2 Emory Healthcare

## Abstract

**Background:** Prosthetic joint infection (PJI) is a serious complication of joint arthroplasty, typically requiring both surgical management and prolonged antimicrobial therapy which has historically been administered via outpatient parenteral antibiotics (OPAT). Recent studies have shown that oral (PO) antibiotics are non-inferior to intravenous (IV) antibiotics in the treatment for PJI and result in fewer catheter related complications. We aimed to examine the impact of a pilot institutional PJI guideline designed to encourage the use of PO antibiotics for PJI treatment on provider ordering practices at a specialty orthopedic hospital. **Methods:** In November 2024, our antimicrobial stewardship committee created and published institutional PJI guidelines which outlined appropriate use of PO antibiotics for PJI. Concurrently, we educated Infectious Diseases (ID) providers who practiced at the hospital. We identified all patients who had an ICD-10 code for prosthetic joint infection (T84.5) in the 12 months pre- (November 2023 – October 2024) and post- (November 2024 – October 2025) guideline implementation. Antibiotic days of therapy (DOT) were defined as the number of days where a patient received 1 or more antibiotics on a given calendar day. Primary outcomes were proportion of patients with OPAT orders and number of Peripherally Inserted Central Cathter (PICC) lines placed. Chi-squared (or when indicated, Fisher’s exact) tests examined proportions of patients who had OPAT orders placed and PICC line orders placed in the pre-guideline period compared with the post-guideline period. Student t-tests compared means for length of stay (LOS), outpatient IV and PO DOT in the pre-guideline period compared to the post-guideline period. **Results:** There were 82 PJI cases identified in the pre-intervention period and 94 in the post-intervention period. There were no differences in the number of OPAT orders placed (30 v. 45, p=0.25), proportion of PICC lines placed [37 (SD 45.1%) v 43 (SD 45.7%), p = 0.25] and mean LOS [5.9 (SD 4.2) v. 6.2 (SD 4.0), p = 0.68] between pre- and post-intervention periods (Table) Outpatient IV DOT [22.5 (SD 58.0) v 22.8 (SD 42.1), p=0.97) and outpatient PO DOT [25.6 (SD 52.9) v 28.0 (SD 35.7), p=0.73) did not significantly change between pre- and post-intervention periods. **Conclusions:** At a specialty orthopedic hospital, implementation of pilot guidelines for PJI was not sufficient to change ID provider ordering practices for PJI treatment. Future work will evaluate the utility of individualized feedback to increase appropriate use of PO antibiotics for PJI.

**Figure:**